# Association of NLRP3 polymorphisms with susceptibility to primary gouty arthritis in a Chinese Han population

**DOI:** 10.1007/s10067-017-3900-6

**Published:** 2017-12-07

**Authors:** Quan-Bo Zhang, Yu-Feng Qing, Yong-Long He, Wen-Guang Xie, Jing-Guo Zhou

**Affiliations:** 1 Department of Geriatrics of the Affiliated Hospital of North Sichuan Medical College, Nanchong, Sichuan 637007 China; 2 Department of Rheumatology and Immunology of the Affiliated Hospital of North Sichuan Medical College, Nanchong, Sichuan 637000 China; 3 Institute of Rheumatology of North Sichuan Medical College, Nanchong, Sichuan 637007 China

**Keywords:** Gouty arthritis, Interleukin1β, mRNA, NLRP3, Polymorphism

## Abstract

The NLRP3-interleukin1β (IL1β) signaling pathway is involved in monosodium urate (MSU)-mediated inflammation. The aim of this present study was to determine whether single nucleotide polymorphisms (SNPs) in the NLRP3 gene are associated with susceptibility to gouty arthritis (GA) and whether these SNPs alter the expression of components of the NLRP3-IL1β signaling pathway. The rs10754558, rs4612666, and rs1539019 SNPs were detected in 583 patients with GA and 459 healthy subjects. NLRP3 and IL1β mRNA levels in peripheral blood mononuclear cells (PBMCs) and serum IL1β levels were measured in different genotype carriers, and correlations between the NLRP3 SNPs and NLRP3 mRNA, IL1β mRNA, and serum IL1β levels were investigated. The GG genotype of NLRP3 rs10754558 was found to be significantly associated with patients with GA compared to the healthy control subjects via multivariate logistic regression analysis (adjusted OR = 2.68, *P* = 0.006). The CGA haplotypes were independently associated with patients with GA compared to the healthy control subjects (adjusted OR = 1.968, *P* = 0.02). The levels of NLRP3 mRNA, IL1β mRNA, and serum IL1β in the patients with GA were significantly different among the three genotypes of rs10754558 (all *P <* 0.01). The GG genotype of rs10754558 and the CGA haplotype of rs4612666-C, rs10754558-G, and rs1539019-A are both independent risk factors for primary GA development. The rs10754558 polymorphism might participate in regulating immune and inflammation responses in patients with GA by influencing the expression of components of the NLRP3 inflammasome. Future multicenter studies aimed at replicating these findings in an independent population as well as functional tests will aid in further defining the role of these SNPs in the development of GA.

## Introduction

Gout arthritis (GA) is one of the most common forms of autoinflammatory arthritis among men and is characterized by elevated urate and monosodium urate (MSU) crystal deposition in tissues, leading to arthritis, soft-tissue masses (i.e., tophi), nephrolithiasis, and urate nephropathy [1]. Although the concrete pathogenesis of gout is still unclear, accumulating evidence indicates that genetic factors, including susceptibility genes that control the production and clearance of urate and lead to hyperuricemia [2], as well as environmental triggers and immune dysregulation might be involved in its development. Meta-analysis of 14 prior studies totaling 28,141 participants demonstrated that genetic polymorphisms of SLC2A9 and URAT1 are key regulators of urate homeostasis, as the inheritance of one predisposing variant of SLC2A9 or URAT1 significantly increases the risk for an individual to develop gout [3]. However, an epidemiological study found that only about 10% of patients with hyperuricemia develop gout [4]. Why certain hyperuricemic individuals are predisposed to gout is not clear; however, this prior study suggests that other genes unrelated to urate metabolism are likely to contribute to the disease susceptibility.

An attack of gout is triggered by the deposition of MSU crystals in the joint, as MSU crystals are recognized as an endogenous danger signal by components of the innate immune system [5]. Pathogen recognizing receptors (PRRs) are major triggers of innate immunity, including toll-like receptors (TLRs) and nod-like receptors (NLRs). Among the PRRs, the intracellular NLRs have been identified as key mediators of inflammatory and immune responses [6, 7].

NLRP3 (NALP3/PYPAF1/Cryoprin/CIASI) belongs to the family of NLR proteins that are comprised of a nucleotide-binding domain and a leucine-rich repeat domain [8]. NLRP3 is expressed predominantly in peripheral blood leukocytes [9]. In response to invading pathogens, NLRP3 rapidly forms a cytoplasmic complex with the apoptosis-associated speck-like protein and the caspase 1 protease, forming the NLRP3 inflammasome. This protein complex plays a central role in the maturation and secretion of the proinflammatory cytokines IL1β and IL18 [6, 7]. Evidence also suggests that genetic variants within the NLRP3 gene might be an important determinant affecting the magnitude of immune inflammatory responses, affecting the susceptibility to infectious and noninfectious diseases [10].

The involvement of the NLRP3 inflammasome in MSU recognition has been previously demonstrated [11, 12]. It has been shown that innate immunity can be activated via the NLRP3 inflammasome in the presence of MSU [5]. Furthermore, stimulation of macrophages with MSU leads to the activation of caspase 1 in an NLRP3-dependent manner, and macrophages deficient in components of the NLRP3 inflammasome are incapable of secreting the proinflammatory cytokine IL1β in response to MSU [11].

In the present study, we evaluated the frequency distribution of three common single nucleotide polymorphisms (SNPs; rs4612666, rs10754558, and rs1539019) within the NLRP3 gene in patients with GA and healthy control individuals and investigated whether these SNPs could be associated with the susceptibility to GA in a Chinese Han population. We also determined whether the SNPs altered the expression of components of the NLRP3-IL11β signaling pathway in peripheral blood from the patients with gout and investigated the clinical relevance of these SNPs in relation to the development of GA.

## Methods

### SNP selection

The SNPs rs4612666 (C/T at intron 7) and rs1539019 (C/A at intron 8) were selected as they have been previously associated with inflammatory disorders [13, 14]. The SNP rs10754558 (C/G) in the 3′ untranslated region (3′-UTR) of the NLRP3 gene was selected because of its recently reported contribution to mRNA stability [15].

### Study population

Two groups of individuals from the same geographical region were compared in this population-based case-control study. All participants were Han (self-reported) from Sichuan, China. A total of 583 consecutive patients with primary GA attending the Department of Rheumatology, the Affiliated Hospital of North Sichuan Medical College, between June 2008 and February 2013 were included in the study. The patients were confirmed to have GA according to the American College of Rheumatology (ACR) classification criteria (1977), which include (1) more than one attack of acute arthritis, (2) maximum inflammation developed within 1 day, (3) oligoarthritis attack, (4) redness observed over joints, (5) first metatarsophalangeal joint painful or swollen, (6) unilateral first metatarsophalangeal joint attack, (7) unilateral tarsal joint attack, (8) tophus (proven or suspected), (9) hyperuricemia, (10) asymmetric swelling within a joint on X-ray, (11) complete termination of an attack [16] and, in addition, had no history of cancer, hematopathy, nephropathy, infection, or other autoimmune diseases. Patients with GA were divided into an acute gouty arthritis (AGA) group and a non-acute gouty arthritis (NAGA) group based on whether they were presented onset of symptoms or not at the joints (including swelling, redness, stiffness, and severe pain). The patients with GA were not receiving any systemic anti-inflammatory treatment or other drugs to control the production and/or elimination of uric acid before blood samples were obtained.

All clinical data were carefully recorded, and a complete medical history was obtained from all patients, including details of previous episodes of GA, any associated systemic diseases, and previous use of anti-inflammatory medications or agents to control the production and/or elimination of uric acid. A total of 459 age-matched male subjects (healthy control subjects) undergoing regular physical examinations at the Affiliated Hospital of North Sichuan Medical College between June 2008 and February 2013 were included as control subjects in this study. The healthy control subjects had no history of systemic inflammatory disease (Table 1). All participants were from a Chinese Han population. Blood samples from all participants were obtained and collected in sterile, anticoagulant-coated tubes and immediately transported to the laboratory for genetic analyses and assessment of gene expression and cytokine production.

**Table 1:** Clinical and demographic characteristics of gout cases and control subjects

	GA group(*n* = 583)	HC group (*n* = 459)	*P* value
Age (years)^a^	48.18 ± 12.16	47.35 ± 11.28	> 0.05
Gender (male/female)^b^	558/25	436/23	> 0.05
Disease duration (years)	6.50 ± 7.30	–	–
Tophi, *n* (%)	71(12.2)	–	–
BMI(kg/m^2^)^a^	25.99 ± 3.32	23.18 ± 4.47	< 0.01
sUA (μmol/L)^a^	509 ± 132.6	308 ± 56.3	< 0.01
GLU (mmol/L)^a^	6.27 ± 1.82	5.18 ± 0.49	< 0.01
WBC (×10^9^/L)^a^	6.89 ± 2.41	4.78 ± 1.55	< 0.01
GR (×10^9^/L)^a^	4.75 ± 2.25	3.48 ± 0.88	< 0.01
LY (×10^9^/L)^a^	1.79 ± 0.73	2.13 ± 0.65	> 0.05
Mo (×10^9^/L)^a^	0.69 ± 0.27	0.49 ± 0.19	< 0.01
TG (mmol/L)^a^	2.34 ± 1.60	1.15 ± 0.67	< 0.01
GLOB (g/L) ^a^	29.96 ± 5.22	26.63 ± 4.31	< 0.01
TC (mmol/L)^a^	4.88 ± 0.98	4.45 ± 0.44	> 0.05
HDL (mmol/L)^a^	1.16 ± 0.41	1.36 ± 0.31	< 0.05
LDL (mmol/L)^a^	2.62 ± 0.87	2.65 ± 0.55	> 0.05
VLDL (mmol/L)^a^	1.13 ± 0.69	0.53 ± 0.22	< 0.01
apoA1 (mmol/L)^a^	1.30 ± 0.35	1.24 ± 0.20	> 0.05
apoB100 (mmol/L)^a^	0.93 ± 0.26	0.78 ± 0.14	< 0.05
ESR	30.26 ± 25.39		
CRP	18.36 ± 26.21		

*GA* gouty arthritis, *HC* healthy control subjects, *BMI* body mass index, *sUA* serum uric acid, *GLU* serum glucose, *WBC* white blood cell counts, *GR* neutrophile granulocytecounts, *LY* lymphocyte counts, *Mo* monocyte counts, *TG* triglycerides, *TC* total cholesterol, *HDL* high density lipoprotein, *LDL* low density lipoprotein, *VLDL* very low density lipoprotein, *apoA1* apolipoprotein A1, *apoB100* apolipoprotein B100, *ESR* erythrocyte sedimentation rate, *CRP* C-reactive protein

^a^ *t* test or corrected *t* test

^b^χ^2^ test were performed. Statistical significance was set at *P* ≤ 0.05

The Ethics Committee of the Affiliated Hospital of the North Sichuan Medical College approved the study protocol, and all participants gave their written informed consent to participation in the study at the time of inclusion and again at the time of follow-up investigations. The study was conducted in accordance with the principles of the current version of the Declaration of Helsinki. The design, analysis, and interpretation of the present study were based on those presented by CM Lewis [17].

### Laboratory examination of regulatory parameters

Plasma total cholesterol (TC), triglycerides (TG), high density lipoprotein cholesterol (HDL), low density lipoprotein cholesterol (LDL), very low density lipoprotein (VLDL), apolipoprotein A1 (apoA1), apolipoprotein B100 (apoB100), and serum uric acid (sUA) were measured using a 7170S auto-analyzer (Hitachi Co, Tokyo, Japan) by an expert who was blinded to the study. The erythrocyte sedimentation rates (ESR), blood cell counts, C-reactive protein (CRP) levels, and serum glucose (GLU) levels were assessed via routine laboratory tests. All of the measurements were performed by the Department of Clinical Laboratory, the Affiliated Hospital of North Sichuan Medical College.

### DNA isolation and genetic analyses

Genomic DNA was isolated from whole blood samples of 583 GA cases and 459 control subjects using a Pure Gene DNA Blood Kit (Gentra, Minneapolis, MN, USA). DNA samples were genotyped using 5′ exonuclease TaqMan® technology (Applied Biosystems, Foster City, CA, USA).

All of the genotyping assays were designed by Applied Biosystems (Foster City, CA, USA).

The genotypes were assigned by an investigator who was blinded to the patients’ clinical status.

The genotyping reaction utilizes two dual-labeled TaqMan probes that specifically target the alternate alleles. The two probes were labeled with a fluorescent reporter dye (VIC or FAM) and a non-fluorescing quencher/minor groove binder (MGB). When a probe specifically binds to the SNP site, the 5′ nuclease activity of the Taq polymerase during the PCR allows for the cleaving and subsequent fluorescence of the reporter dye. At the conclusion of the PCR, the samples were genotyped by analysis of the fluorescence of the two dyes. Each 5.0 μL PCR contained the following: TaqMan® Universal PCR Master Mix, No AmpErase® UNG (2×), Assays-on-Demand™ (20×) or Assays-by-Design™ (40×) SNP Genotyping Assay Mix, and 1 ng of genomic DNA. Assays were conducted in a 96-well format on the ABI PRISM® 7900HT Sequence Detection System (Applied Biosystems, Foster City, CA, USA). Reaction conditions were the following: initial denaturation at 95 °C for 10 min, followed by 40 cycles each of denaturation (92 °C for 15 s), and annealing/extension (60 °C for 60 s). Case and control DNA was genotyped together on the same plates with duplicate samples (15%) to assess intraplate variation and interplate genotype quality. No genotyping discrepancies were detected. These methods were performed according to a previous study [18].

### RNA extraction and real-time quantitative PCR amplification of the NLRP3 and IL1β genes

PBMCs were isolated using Ficoll-Hypaque density gradient centrifugation from blood samples of 135 patients with AGA, 135 patients with NAGA, and 108 healthy subjects. Total RNA was extracted using Trizol reagent (Invitrogen, Carlsbad, CA, USA) from PBMCs then reverse-transcribed into cDNA using reagents that included a random hexamer, superscript II, and dNTP (Invitrogen, USA). The converted cDNA was cryopreserved at − 80 °C until real-time quantitative PCR (RT-qPCR) was performed.

RT-qPCR was carried out at a final volume of 20 μL in an ABI Prism 7900HT Sequence Detection System (Applied Biosystems, USA). The reaction contained Power SYBR Green PCR Master Mix (Applied Biosystems, USA; 9 μL), 10 pmol/L each of forward and reverse primers (0.5 μL each), synthesized cDNA sample (1.3 μL), and ddH_2_O (8.7 μL). The thermal cycling conditions comprised of an initial denaturing step at 95 °C for 10 min, 40 cycles of renaturation at 95°C for 15 s, and elongation at 60°C for 1 min. The PCR reaction for each gene was duplicated for each sample, and the mean value was used for further analysis. Additionally, the RT-qPCR reaction was run according to a modification of the Cawthon method [19, 20]. The sequences of the primers used for PCR are given in Table 2.

**Table 2:** Sequences of primers used in the RT-qPCR assays

Gene	Forward primer	Reverse primer
NLRP3	5′-CCCCGTGAGTCCCATTA-3′	5′-GACGCCCAGTCCAACAT-3′
IL1β	5′-GAGCTACGAGCTGCCTGACG-3′	5′-GTAGTTTCGTGGATGCCACAG-3′
β-Actin	5′-GAGCTACGAGCTGCCTGACG-3′	5′-GTAGTTTCGTGGATGCCACAG-3′

We used relative quantification to evaluate the expression of selected genes [19], and the housekeeping gene β-actin was used as an internal control to normalize the mRNA expression of each target gene.

## ELISA

After centrifugation, serum samples were stored at − 80 °C. IL1β levels in the serum obtained from 135 AGA cases, 135 NAGA cases, and 108 healthy subjects were determined using enzyme immunoabsorbent assay (ELISA) kits (R&D Systems, Minneapolis, MN, USA). The detection limits of the IL1β assay were 5–200 pg/mL. The optical density was determined using a microplate reader (Model 3550; Bio-Rad, Hercules, CA, USA). A standard curve for cytokine IL1β was established using a known concentration of IL1β by plotting the optical density relative to the log of the concentration.

### Statistical analysis

Statistical analyses were performed using SPSS 16.00 (SPSS, Chicago, IL, USA) and the Haploview software [21]. The genotype distribution was analyzed for deviations from the Hardy-Weinberg equilibrium (HWE) using χ^2^ analyses. Differences in the allele and genotype frequencies between the case and control subjects were assessed using the χ^2^ test with a 3 × 2 or 2 × 2 contingency. Linkage disequilibrium and haplotype analyses were also performed using Haploview software. Associations between genotypes and GA were estimated by computing the odds ratios (OR) and their 95% confidence intervals (CI) from multivariate logistic regression analysis with adjustments for alcohol consumption, dietary factors, hypercholesterolemia, hypertension, hyperuricemia, BMI, and gender, all of which represent possible confounders. One-way analysis of variance (ANOVA) in conjunction with the LSD method for multiple comparisons was performed. All the statistical tests were two-sided at a significance level of 0.05. The statistical power was calculated using PS software (http://biostat.mc.vanderbilt.edu/twiki/bin/view/Main/PowerSampleSize).

## Results

### Clinical and laboratory characteristics of the study subjects

The clinical and laboratory data of the subjects are summarized in Table 1. In our cohorts, gout cases were matched for age and gender to the control individuals (all *P* > 0.05). In total, 71 (12.2%) of the 583 patients with GA had tophi. Significant differences were observed between the GA and HC groups, including the levels of BMI, WBC, GR, Mo, GLOB, sUA, GLU, TG, VLDL, HDL-C, and apoB100 (all *P* < 0.05). WBC, GR, Mo, sUA, BMI, GLU, TG, VLDL, apoB100, and GLOB were significantly increased in patients with GA compared to HC subjects (all *P* < 0.05 or *P* < 0.01; Table 1).

### Association of NLRP3 SNPs genotypes and alleles with GA risk

The genotyping success rate was 100% in this study. The primary information and allele frequencies observed are listed in Table 3. All genotyped distributions of case and control subjects were consistent with those expected from the Hardy-Weinberg equilibrium, except for rs1539019 in control subjects. The minor allele frequency (MAF) of all three SNPs was consistent with that reported in the HapMap database (http://www.hapmap.org).

**Table 3:** Primary information of genotyped SNPs

Rs no.	Location	Base	MAF		HWE	
			Case	Control	Case	Control
rs10754558	3′ UTR	G > C	0.452	0.388	0.605	0.698
rs4612666	Intron 7	T > C	0.449	0.429	0.311	0.424
rs1539019	Intron 8	A > C	0.420	0.392	0.646	0.026

*MAF* minor allele frequency, *HWE* ^*P*^ *P* value of Hardy-Weinberg equilibrium (HWE)

Significant differences were observed between the GA and HC groups with respect to the genotype and allele frequencies of rs10754558 (χ^2^ = 9.103, 8.66, *P* = 0.011, 0.003, respectively). As shown in Table 4, the frequencies of the GG genotype and G allele were significantly increased in the GA group compared with the HC group (all *P* < 0.05; Table 4). With the current sample size (583 GA and 459 healthy subjects as controls), an OR of 1.30 with an exposure frequency of 38.8% was detected with 84.3% power at a significance level of 0.05. Individuals carrying the GG genotype had a higher risk for developing gout than those carrying the CC genotype (*P* = 0.006, OR = 1.653, adjusted OR = 2.66, 95% CI 1.01–7.03; Table 4), and individuals carrying the G allele had a higher risk for developing gout than those carrying the C allele (*P* = 0.003, OR = 1.302, 95% CI 1.092–1.552; Table 4).

**Table 4:** Distributions of genotypes of the NLRP3 gene rs10754558 and their associations with the risk of GA

Variables	GA (*n* = 583)	HC (*n* = 459)	χ^2^	*P*	OR (95% CI)	OR (95% CI)^A^
	*n* (%)	*n* (%)				
CC^Reference^	172 (29.5%)	174 (37.9%)	–	–	1.00	1.00
CG	295 (50.6%)	214 (46.6%)	5.65	0.017	1.40 (1.06–1.84)	1.09 (0.56–2.36)
GG	116 (19.9%)	71 (15.5%)	7.48	0.006	1.65 (1.15–2.38)	2.68 (1.13–7.26)
C allele^Reference^	639 (54.8%)	562 (61.2%)	–	–	1.00	
G allele	527 (45.2%)	356 (38.8%)	8.66	0.003	1.30(1.06–1.84)	

*GA* gouty arthritis, *HC* healthy subjects, *OR* odds ratio, *CI* confidence interval

^A^Adjusted for alcohol consumption, dietary factors, hypercholesterolemia, hyperuricemia, hypertension, BMI, and gender in logistic regression model

The frequencies of the CC, CT, and TT genotypes of NLRP3 rs4612666 in the GA and HC groups were 0.299/0.504/0.197 and 0.303/0.536/0.161, respectively. The frequencies of the C/T allele in the GA and HC groups were 0.551/0.449 and 0.571/0.429, respectively. The frequencies of the CC, CA, and AA genotypes of NLRP3 rs1539019 in the GA and HC groups were 0.166/0.508/0.326 and 0.153/0.479/0.368, respectively. The frequencies of the C/A allele in the GA and HC groups were 0.42/0.58 and 0.392/0.608, respectively. No significant differences were observed between the GA and HC groups with respect to genotype and allele frequencies of rs4612666 and rs1539019 (rs4612666: χ^2^ = 2.35, 0.85; *P* = 0.31, 0.36, respectively; rs1539019: χ^2^ = 2.06, 0.32; *P* = 0.36, 0.39, respectively).

### Association of NLRP3 SNP haplotypes with GA risk

D’ values and r^2^ values form linkage disequilibrium (LD) showed that the D’ values between rs10754558 and rs4612666, rs10754558 and rs1539019, and rs4612666 and rs1539019 are 0.812, 0.817, and 0.803, respectively, the r^2^ values between rs10754558 and rs4612666, rs10754558 and rs1539019, and rs4612666 and rs1539019 are 0.346, 0.378, and 0.309, respectively. The data suggested that three of the NLRP3 SNP loci (rs4612666, rs10754558, and rs1539019) were in LD (D’ > 0.8 and r^2^ > 0.3 are considered significant LD). The common haplotypes were CCC, CCA, CGC, CGA, TCA, TGC, and TGA, accounting for 98.3% of the haplotypes observed in all of the individuals studied (Table 5). The frequency of the CCC and TCA haplotypes in GA cases was only slightly lower than that in the HC subjects (all *P* > 0.05; Table 5), while the frequency of the CGC, TGC, and TGA haplotypes was only slightly higher in GA cases than in the HC subjects (all *P* > 0.05; Table 5). The frequency of the CCA haplotype was significantly decreased in GA cases compared with the HC subjects (*P* < 0.05; Table 5), while the frequency of the CGA haplotype was significantly increased (*P* < 0.05; Table 5). The risk for developing GA was significantly increased in individuals carrying the CGA haplotype (adjusted OR = 1.920, *P* = 0.02; Table 5) and decreased in individuals with the CCA haplotype (adjusted OR = 0.580, *P* = 0.001; Table 5).

**Table 5:** Distributions of haplotypes of the NLRP3 gene and their associations with risk of GA

Haplotypes	GA (*n* = 583)	HC (*n* = 459)	χ^2^	*P*	OR (95% CI)	OR (95% CI)^A^
	*n* (%)	*n* (%)				
CCC	43 (7.4)	34 (7.5)	0.003	0.954	0.990 (0.650–1.501)	0. 868 (0.462–1.216)
CCA^*^	92 (15.7)	106 (23.1)	11.49	0.001	0.620 (0.472–0.820)	0.565 (0.366–0.768)
CGC	136 (23.4)	97 (21.1)	0.99	0.320	1.140 (0.877–1.493)	1.009 (0.588–1.193)
CGA^*^	54 (9.3)	26 (5.7)	5.42	0.020	1.680 (1.081–2.619)	1.968 (1.18–3.453)
TCA	176 (30.2)	141 (30.7)	0.029	0.865	0.980 (0.770–1.240)	0.986 (0.765–1.132)
TGC	51 (8.8)	38 (8.2)	0.145	0.703	1.080 (0.730–1.60)	0.766 (0.658–1.218)
TGA	20 (3.5)	10 (2.2)	2.018	0.155	1.660 (0.820–3.340)	1.268 (0.763–2.189)

All frequencies < 0.03 are ignored

*GA* gouty arthritis, *HC* healthy subjects, *OR* odds ratio, *CI* confidence interval

^A^Adjusted for alcohol consumption, dietary factors, hypercholesterolemia, hyperuricemia, hypertension, BMI, and gender in the logistic regression model

### Association of the rs10754558 polymorphism with NLRP3 mRNA levels and serum IL1β production

**Fig. 1:**
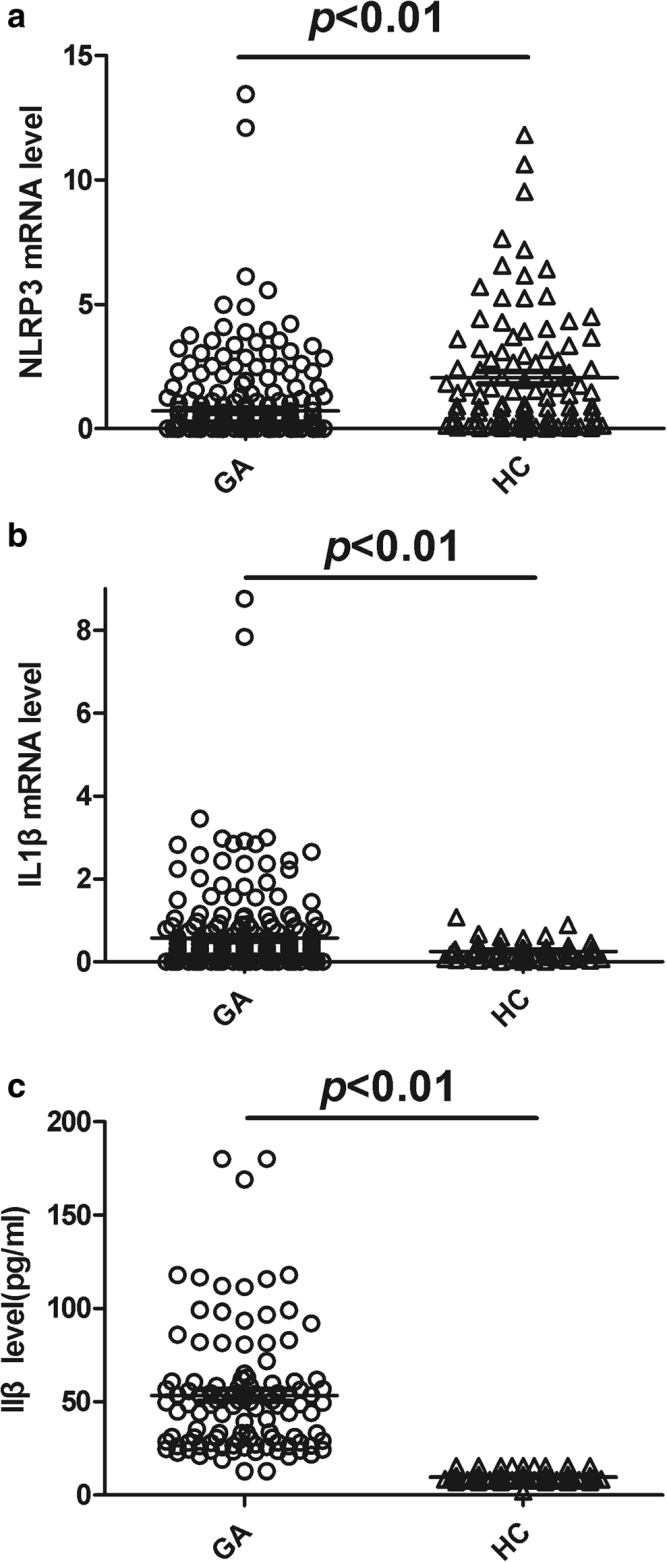
Levels of NLRP3 and IL1β mRNAs in PBMCs and serum IL1β in the GA and HC groups. **a** Expression of NLRP3 mRNA in PBMCs for the 270 patients with GA and 108 healthy subjects was detected using RT-qPCR. NLRP3 mRNA levels were significantly decreased in the GA group compared with the HC group (*P* < 0.01). **b**, **c** Expression of the IL1β mRNA in PBMCs and serum IL1β were measured in the 270 patients with GA and 108 healthy subjects. Expression of the IL1β mRNA and serum IL1β levels were much higher in the patients with GA than those in the HC group (*P* < 0.01; respectively). GA, gouty arthritis; HC, healthy controls. The Kruskal-Wallis’s test was performed; statistical significance was set at *P* ≤ 0.05

The level of the NLRP3 mRNA in PBMCs was significantly decreased in the GA group compared with the HC group (*P* < 0.01; Fig. 1a), whereas the levels of the IL1β mRNA in PBMCs and serum IL1β were significantly increased in the GA group compared with the HC group (all *P* < 0.01; Fig. 1b, c). The levels of NLRP3 mRNA, IL1β mRNA, and serum IL1β in patients with AGA significantly differed among the three genotypes of rs10754558 (*F* = 13.55, 30.44, 65.33, *P <* 0.0001; respectively). NLRP3 mRNA and IL1β mRNA levels as well as serum IL1β levels from patients with NAGA also significantly differed among the three genotypes (*F* = 15.32, 23.61, 44.64, *P <* 0.0001; respectively). There were no differences in NLRP3 mRNA and IL1β mRNA levels as well as serum IL1β levels between the three different genotypes in healthy subjects (all *P* > 0.05).

**Fig. 2:**
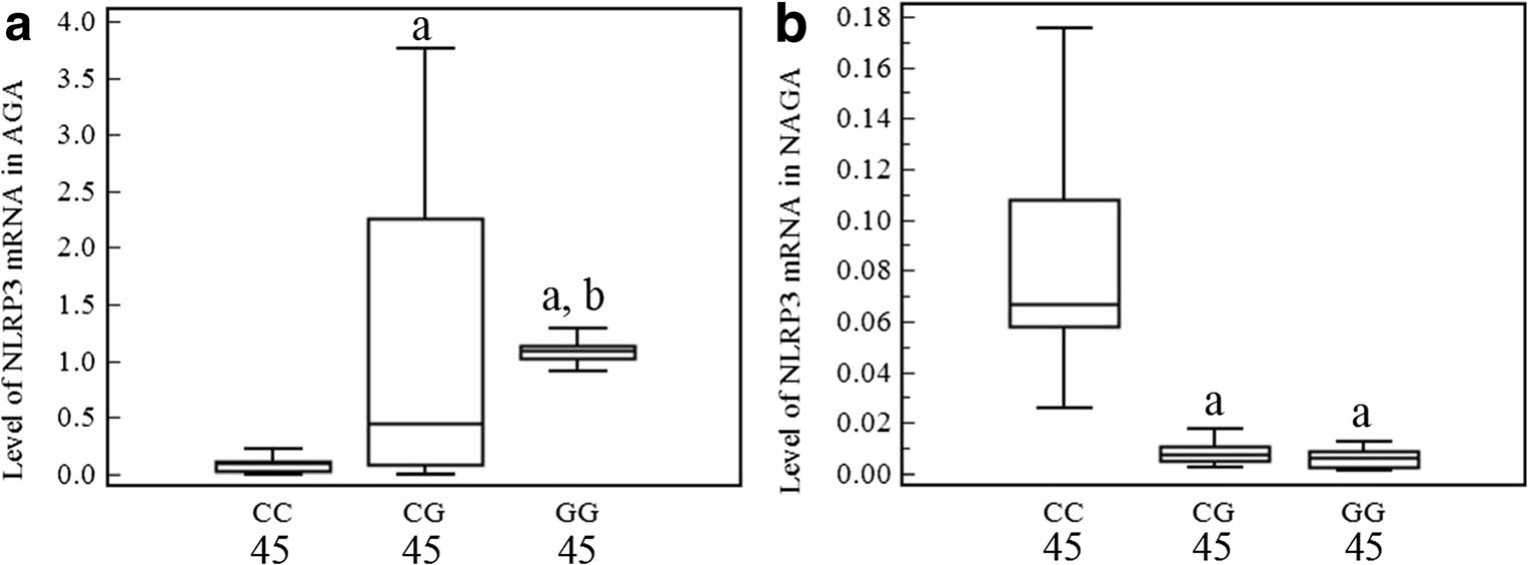
Association of the NLRP3 rs10754558 SNP with NLRP3 mRNA levels in PBMCs from patients with GA. The levels of NLRP3 mRNA in PBMCs were measured in the different genotype carriers from the GA group. The levels of the NLRP3 mRNA differed significantly among the three genotypes of rs10754558, both in patients with acute and non-acute GA (*F* = 13.55, 15.32, all *P <* 0.0001). **a** In patients with acute GA, the NLRP3 mRNA expression levels in 45 GG homozygotes and 45 heterozygotes carriers was significantly increased compared with 45 CC homozygotes carriers (all *P* < 0.01), and NLRP3 mRNA levels were higher in GG homozygotes carriers than that in CG heterozygotes carriers (*P* < 0.01). **b** In patients with non-acute GA, the NLRP3 mRNA levels were significantly reduced in the 45 GG homozygotes or 45 GT heterozygotes carriers compared with 45 CC homozygotes carriers (all *P* < 0.01); no difference was observed between the GG and CG genotypes (*P* > 0.05). The data are shown as box plots. Each box represents the upper and lower interquartile range (IQR). The whiskers represent 1.5 times the upper and lower IQRs. The ANOVA, LSD method was performed. ^a^ *P* < 0.01 in comparison with patients with the CC genotype; ^b^ *P* < 0.01 in comparison with patients with the CG genotype. The statistical significance was set at *P* ≤ 0.05

**Fig. 3:**
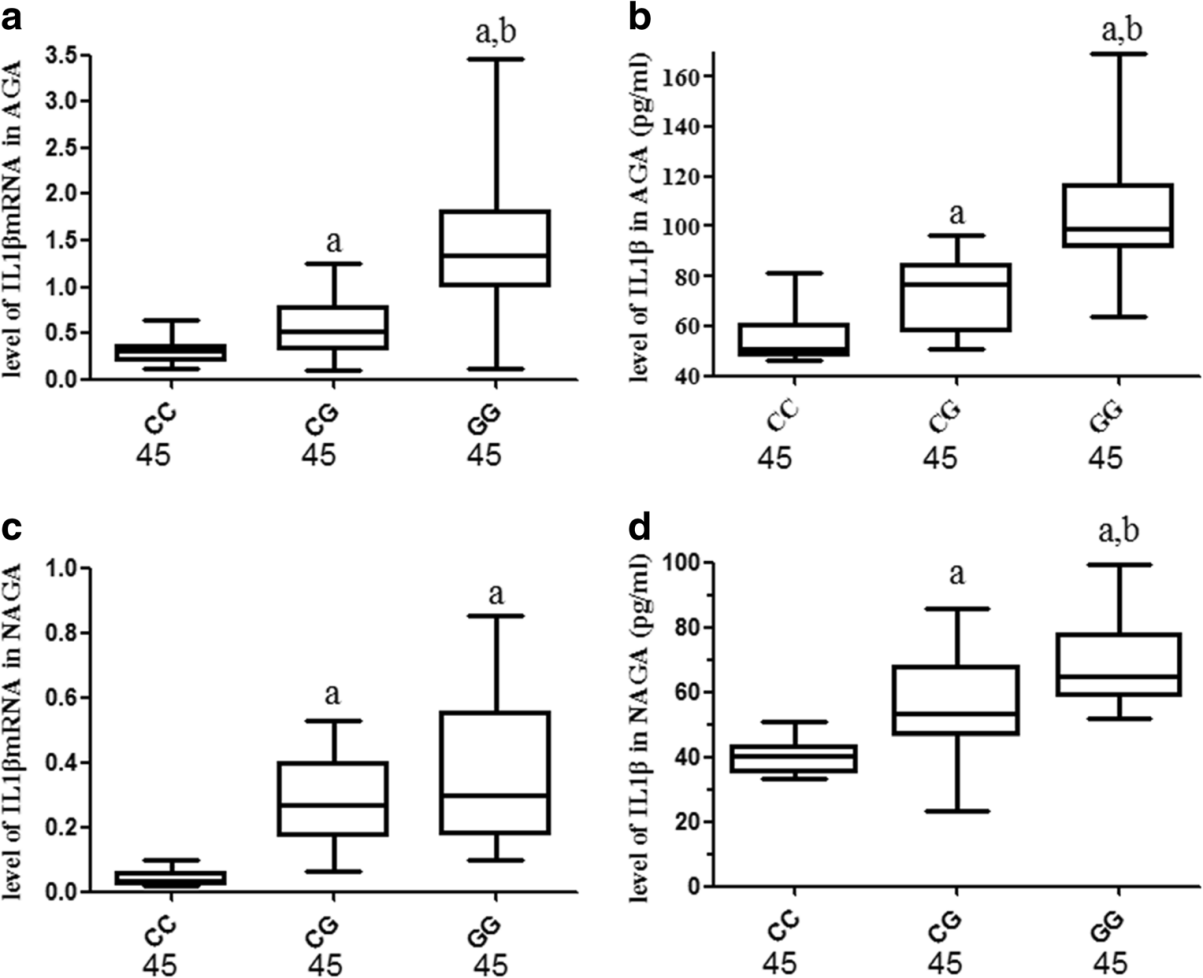
Association of the NLRP3 rs10754558 polymorphism with IL1β mRNA levels in PBMCs and serum IL1β levels from patients with GA. The levels of IL1β mRNA in PBMCs and IL1β in serum were measured in different genotype carriers from patients with GA. Significant differences were observed among the three genotypes of rs10754558 with respect to IL1β mRNA and serum IL1β levels both from patients with acute and non-acute GA (IL1β mRNA: *F* = 30.44, 23.61, all *P <* 0.0001, respectively; IL1β: *F* = 65.33, 44.64, all *P <* 0.0001, respectively). **a**, **b** In patients with acute GA, the IL1β mRNA and serum IL1β levels in the 45 GG homozygotes and 45 heterozygotes carriers were significantly increased compared with 45 CC homozygotes carriers (all *P* < 0.01), and IL1β mRNA and serum IL1β levels were higher in GG homozygotes carriers than in CG heterozygotes carriers (all *P* < 0.01). **c** In patients with non-acute GA, IL1β mRNA levels were much higher among the 45 GG and 45 CG genotype carriers than in the 45 CC genotype carriers (all *P* < 0.01); no difference was observed between the GG and CG genotypes (*P* > 0.05). **d** In patients with non-acute GA, serum IL1β levels in the 45 GG homozygotes and 45 heterozygotes carriers was significantly increased compared with the 45 CC homozygotes carriers (all *P* < 0.01), and serum IL1β levels were higher in the GG homozygotes carriers than in the heterozygotes carriers (*P* < 0.01). The data are shown as box plots. Each box represents the upper and lower interquartile range (IQR). The whiskers represent 1.5 times the upper and lower IQRs. The ANOVA, LSD method was performed. ^a^ *P* < 0.01 in comparison with patients with the CC genotype; ^b^ *P* < 0.01 in comparison with patients with the CG genotype. The statistical significance was set at *P* ≤ 0.05

As shown in Fig. 2, NLRP3 mRNA expression levels in PBMCs from GG homozygotes and heterozygotes from the patients with AGA were significantly higher than in the CC homozygotes (all *P* < 0.01; Fig. 3a) and higher in the GG homozygotes carriers than in the CG homozygotes (*P* < 0.01; Fig. 2a). However, in the patients with NAGA, NLRP3 mRNA levels in PBMCs were significantly reduced in the GG homozygotes and heterozygotes carriers compared with the CC homozygotes (*P* < 0.01; Fig. 2b); no difference was observed between the CG and GG genotypes (*P* > 0.05; Fig. 2b). As shown in Fig. 3, IL1β mRNA levels in PBMCs from the patients with AGA and serum IL1β production from the patients with AGA or NAGA among the GG homozygote and heterozygote carriers were significantly increased compared with the CC homozygote carriers (all *P* < 0.05; Fig. 3a, b, d) and higher in the GG genotype carriers than that in the CG carriers (*P* < 0.01; Fig. 3a, b, d). Among the patients with NAGA, IL1β mRNA levels in PBMCs among the GG and CG genotypes carriers were also much higher than those of the CC carriers (*P* < 0.01; Fig. 3c), but no significant difference was observed between the GG and CG carriers (*P* > 0.05; Fig. 3c).

## Discussion

Identification of the NLRP3 inflammasome as having a role in the recognition of MSU crystals and the subsequent release of IL-1β was a milestone in understanding the pathogenesis of gout [22], and this pivotal role of the NLRP3 inflammasome presents an attractive target for the potential treatment of gout. The present study is, to our knowledge, the first to demonstrate an association between the common NLRP3 rs10754558 polymorphism and GA susceptibility in a Chinese population as based on case-control association analysis.

Recent findings have underscored the relation of NLRP3 SNPs and some inflammatory diseases susceptibilities [23–34]. With respect to the associated risk of GA in a Chinese Han population, we investigated three tag SNPs of the NLRP3 gene in this present study. The frequencies of the GG genotype and the G allele of rs10754558 were significantly increased in patients with GA compared with healthy controls. Our results indicated that the GG genotype and the G allele of rs10754558 significantly increase the risk of GA in this Chinese Han population. Adjusting multivariate logistic regression analysis for alcohol consumption, dietary factors, hypercholesterolemia, hyperuricemia, hypertension, BMI, and gender increased the significance of the observed association. Hence, the effect of rs10754558 on susceptibility to gout appears to be independent of common risk factors. To the best of our knowledge, ours is the first study to demonstrate the association of common SNPs in NLRP3 inflammasome genes with an increased risk of GA.

We found that the rs4612666 and rs1539019 SNPs were not associated with GA susceptibility, but the three NLRP3 SNPs were found to be in LD. Haplotype analysis of these three NLRP3 SNPs showed that haplotype CGA (which includes the rs10754558 G allele) occurs more frequently in patients with GA than in the controls. Our results suggested that the haplotype CGA significantly increased the risk of GA in this Chinese Han population, and adjusting multivariate logistic regression analysis for compound factors increased the significance of this association. Thus, we concluded that NLRP3 gene haplotypes (including rs10754558) are likely associated with an increased susceptibility to gout and three NLRP3 SNPs might participate in development of gouty arthritis collectively.

GA is a chronic autoinflammatory disease involving complex interactions among various environmental and genetic factors [35, 36]. Although the pathophysiological mechanisms involved in the development of GA have not been fully identified, different genetic factors [3, 37, 38] and activation of innate immunity via NLRP3 inflammasome sensing of MSU [11, 21] are likely important. An attack of gout is triggered by the deposition of MSU crystals in the joint, and MSU crystals are widely recognized as an endogenous danger signal by components of the innate immune system [5, 6]. It was recently demonstrated that the G allele of the rs10754558 polymorphism enhanced NLRP3 mRNA stability [15]. Therefore, polymorphisms in NLRP3 inflammasome components could result in an unknown pathologic defect and contribute to a genetic background that is more susceptible to GA. However, the exact molecular mechanisms underlying the role of the NLRP3 inflammasome in GA inflammation are not completely understood and still require further investigation.

In this study, NLRP3 mRNA levels in PBMCs were significantly decreased in the GA group compared with the HC group, while the IL1β mRNA levels in PBMCs and IL1β levels in serum were significantly increased in the GA group compared with the HC group, suggesting that NLRP3 inflammasome signaling was activated in the peripheral blood of the patients with GA. In those patients with AGA, NLRP3 mRNA levels in PBMCs were significantly increased for the GG and CG genotype carriers compared with CC genotype carriers, and higher in the GG genotype carriers compared to the CG genotype carriers; while in patients with NAGA, NLRP3 mRNA levels were significantly reduced in the GG genotype carriers relative to the CC genotype carriers. IL1β mRNA levels in PBMCs and IL1β levels in serum were significantly increased in the GG genotype carriers compared with the CC genotype carriers both in patients with AGA and those with NAGA. These results suggested that in patients with AGA the GG genotype of rs10754558 is associated with higher NLRP3 mRNA levels in PBMCs compared with the GG genotype and that the GG genotype is associated with decreased NLRP3 mRNA expression in patients with NAGA. These results indicated that the NLRP3 rs10754558 polymorphism might be involved in the regulation of NLRP3 mRNA expression and IL1β levels in patients with GA. It is noteworthy that the NLRP3 mRNA expression in patients with acute and non-acute GA demonstrates an inverse association. This suggests there might be a regulatory mechanism involving negative feedback in cases of non-acute gout. Although we have found no reports that a polymorphism differentially regulates mRNA expression according to the disease condition, we can not exclude this possibility. Future studies with larger simple sizes and functional tests are required to confirm our results. Nevertheless, it is worth noting that no differences in NLRP3 mRNA, IL1β mRNA, or serum IL1β levels were observed in the peripheral blood from the different genotypes in healthy individuals. Thus, we speculate that the rs10754558 SNP might alter NLPR3 mRNA expression only when NLRP3-IL1β signaling is activated; however, a specific mechanism is not apparent.

In conclusion, our findings indicated that the NLRP3 rs10754558 SNP and its haplotypes were associated with an increased susceptibility to gout. The GG genotype and G allele of rs10754558 were associated with higher NLRP3 and IL1β mRNA levels in PBMCs and increased serum IL1β production; as such, rs10754558 might contribute to the development of GA inflammation by regulating the expression of components of the NLRP3-IL11β signaling pathway. Despite hyperuricemia is the biochemical basis of gout development, only about 5–10% of people with hyperuricemia develop gout. It suggested that genes which regulate inflammation and immunology such as NLRP3, except for genes which regulate urate homeostasis such as SLC2A9, were involved in the pathogenesis of gout. It has been confirmed that NLRP3 inflammasome participated the development of gouty arthritis. Our study found the NLRP3 rs10754558 SNP and its haplotypes were associated with an increased susceptibility to gout; it may partly explain why less 10% of people with hyperuricemia develop gout finally. So, the present study is novelty and interesting. The present experiment is a single center study, and lack of functional experiments to confirm the role of NLRP3 SNPs in gout. To confirm our preliminary results, multicenter studies designed to replicate these findings in an independent population as well as functional tests are needed.
